# Acute Hypoxia Induced an Imbalanced M1/M2 Activation of Microglia through NF-κB Signaling in Alzheimer’s Disease Mice and Wild-Type Littermates

**DOI:** 10.3389/fnagi.2017.00282

**Published:** 2017-08-25

**Authors:** Feng Zhang, Rujia Zhong, Song Li, Zhenfa Fu, Cheng Cheng, Huaibin Cai, Weidong Le

**Affiliations:** ^1^Liaoning Provincial Center for Clinical Research on Neurological Diseases, The First Affiliated Hospital, Dalian Medical University Dalian, China; ^2^Liaoning Provincial Key Laboratory for Research on the Pathogenic Mechanisms of Neurological Diseases, The First Affiliated Hospital, Dalian Medical University Dalian, China; ^3^Laboratory of Neurogenetics, National Institute on Aging, National Institutes of Health Bethesda, MD, United States; ^4^Collaborative Innovation Center for Brain Science, The First Affiliated Hospital, Dalian Medical University Dalian, China

**Keywords:** hypoxia, Alzheimer’s disease, neuroinflammation, microglia, M1/M2 phenotypes

## Abstract

Alzheimer’s disease (AD) is the most common neurodegenerative disease mainly caused by abnormal tau phosphorylation, amyloid β (Aβ) deposition and neuroinflammation. As an important environmental factor, hypoxia has been reported to aggravate AD via exacerbating Aβ and tau pathologies. However, the link between hypoxia and neuroinflammation, especially the changes of pro-inflammatory M1 or anti-inflammation M2 microglia phenotypes in AD, is still far from being clearly investigated. Here, we evaluated the activation of microglia in the brains of APP^swe^/PS1^dE9^ transgenic (Tg) mice and their wild type (Wt) littermates, after a single episode of acute hypoxia (24 h) exposure. We found that acute hypoxia activated M1 microglia in both Tg and Wt mice as evidenced by the elevated M1 markers including cluster of differentiation 86 (CD86), tumor necrosis factor-α (TNF-α), interleukin-6 (IL-6), C-C motif chemokine ligand 2 (CCL2) and CCL3. In addition, the markers of M2 microglia phenotype (arginase-1 (Arg-1), CD206, IL-4 and IL-10) were decreased after acute hypoxia exposure, suggesting an attenuated M2 phenotype of microglia. Moreover, the activation of microglia and the release of cytokines and chemokines were associated with Nuclear factor-κB (NF-κB) induction through toll-like receptor 4 (TLR4). In summary, our findings revealed that acute hypoxia modulated microglia M1/M2 subgroup profile, indicating the pathological role of hypoxia in the neuroinflammation of AD.

## Introduction

Neuroinflammation plays pivotal roles in various neurodegenerative diseases, including Alzheimer’s disease (AD). However, whether it is protective or harmful is still under debate. Although immune response is intended to be protective, excessive inflammatory response may cause tissue damage (Calsolaro and Edison, 2016). Senile plaques composed of amyloid-β (Aβ) polypeptides and neurofibrillary tangles (NFTs) made by abnormally phosphorylated tau proteins are pathological hallmarks of AD. In AD brain, Aβ and NFTs can directly cause neuronal damage and cell death. Indirectly, Aβ and NFTs can also activate immune response and lead to the release of inflammatory cytokines, chemokines, and neurotoxins including reactive oxygen species (ROS), nitric oxide (NO), and excitatory amino acids, which may contribute to the neuronal degeneration. Besides their neurotoxic effects, pro-inflammatory cytokines, such as interleukin-1β (IL-1β), tumor necrosis factor-α (TNF-α), and activated glial cells are believed to promote Aβ production (Azizi et al., 2014; Calsolaro and Edison, 2016). Senile plaques deposition is a result of imbalanced Aβ production and removal. Clearance of Aβ is a complex process mediated by various cellular machinery, including engulfment and degradation by resident microglia and infiltrating innate immune cells. In AD brain, aging and toxic conditions favor the chronic activation of microglia and reduce their phagocytic capacity and prolong neuroinflammation (Zuroff et al., 2017).

Microglia and astrocytes are the main immune cells in the central nervous system (CNS). As the resident macrophages of CNS, microglial cells act as the first and main form of active immune defense (Filiano et al., 2015). Activated microglia could execute many functions such as phagocytosis of toxic products, releasing of cytokines, promotion of repair and antigen-presenting (Morales et al., 2014). It is generally considered that microglia have two different phenotypes of activation: pro-inflammatory M1 and immunosuppressive M2. M1 phenotype, a classical activation, is associated with massive inflammatory response releasing IL-1β, TNF-α and expressing inducible nitric oxide synthase (iNOS). M2 includes the states of both alternative activation and acquired deactivation with an anti-inflammatory profile. Alternative activation responds to IL-4 or IL-13 and promotes resolution of inflammation and tissue repair. Acquired deactivation results from uptake of apoptotic cells or exposure to anti-inflammatory cytokines such as IL-10 and transforming growth factor-β (TGF-β) and alleviates acute inflammation (Orihuela et al., 2016; Tang and Le, 2016). In AD, microglia surrounding and infiltrating the Aβ plaques are generally activated as YM-1 positive M2 phenotype (Jimenez et al., 2008). Pro-inflammatory cytokines, such as interferon-γ (IFN-γ), IL-1β and TNF-α, which shift microglia to M1 activation, attenuate the phagocytosis of Aβ (Koenigsknecht-Talboo and Landreth, 2005). For misfolded tau protein, pro-inflammation cytokines can affect the tau pathological metamorphosis, increasing tau phosphorylation and accelerating tangle formation (Zilka et al., 2012).

Hypoxia, as one of the environmental risk factors, was reported to contribute to the pathogenesis of AD (Zhang and Le, 2010). Previous studies have indicated that hypoxia may increase Aβ production (Li et al., 2009), decrease Aβ degradation (Wang et al., 2011) and enhance tau phosphorylation (Gao et al., 2013; Yagishita et al., 2017), thereafter may further aggravate the pathological changes of AD. Hypoxia is also reported to be associated with neuroinflammation. The results of chronic hypoxia studies showed that intermittent hypoxia increased pro-inflammatory cytokines in microglia and in dorsal hippocampus of mice (Smith et al., 2013; Sapin et al., 2015). These results imply that neuroinflammation may be one of the mechanisms of hypoxia-induced cognitive impairment. Here, we determined the activation status of microglia and the secretion of cytokines after acute hypoxia exposure, and investigated the possible transition of M1/M2 phenotypes in AD mouse model.

## Materials and Methods

### Animals and Hypoxic Treatment

Adult male APP^swe^/PS1^dE9^ transgenic (Tg) mice at the age of 6 months and their age-matched wild-type (Wt) littermates were included. Tg mice were purchased from the Jackson Laboratory (No. 004462, Bar Harbor, MA, USA). All the mice were housed under the condition of controlled light (12 h/12 h light/dark cycle), constant room temperature 22 ± 1°C and relative humidity 50 ± 10%. The mice were randomized into four groups: Tg with acute hypoxia (H-Tg), Tg with normoxia (N-Tg), Wt with acute hypoxia (H-Wt), Wt with normoxia (N-Wt), with 10 mice in each group. The hypoxia groups were exposed to a continued hypoxic condition (oxygen 7%) in a hypoxic chamber for 24 h. The normoxia groups were kept in a similar chamber with normoxic condition. After hypoxic exposure, the mice were immediately sacrificed for pathological and biochemical tests. Animal care and procedures were carried out in accordance with the Laboratory Animal Care Guidelines approved by the Institutional Animal Care Committee at Dalian Medical University. The protocol was approved by the Institutional Animal Care Committee at Dalian Medical University.

### Gene Expression

Protocols for total RNA extraction, cDNA synthesis and quantitative real-time polymerase chain reaction (PCR) were described previously (Tang et al., 2014). Mice were sacrificed immediately after the 24-h hypoxia episode. Bilateral hippocampus (*n* = 4 in each group) were dissected and extracted for total RNA with RNAiso Plus (Total RNA extraction reagent; Takara, Shiga, Japan). According to Revertra Ace qPCR RT kit (Takara, Shiga, Japan) instructions, total RNA was synthesized to cDNA. Real-time PCR was performed with TransStart Top Green qPCR SuperMix (TransGen Biotech, Beijing, China) and monitored by the Real-time PCR System (Applied Biosystems 7500 Real-Time PCR Systems). The primer sequences were provided upon request as summarized in Table 1. The relative expression levels of each primer sequences mRNA were analyzed by the 2^−ΔΔCt^ algorithm normalizing to GAPDH and relative to the control groups.

**Table 1:** Primer sequences for real-time polymerase chain reaction (PCR).

	Forward (5′ to 3′)	Reverse (5′ to 3′)
GAPDH	TGTGTCCGTCGTGGATCTGA	TTGCTGTTGAAGTCG CAGGAG
IL-1β	AAGGGGACATTAGGCAGCAC	ATGAAAGACCTCAG TGCGGG
TNF-α	CCAGTGTGGGAAGCTGTCTT	AAGCAAAAGAGGAG GCAACA
TGF-β	CACTCCCGTGGCTTCTAGTG	CTTCGATGCGCTTC CGTTTC
CCL2	GCATCCACGTGTTGGCTCA	CTCCAGCCTACTCATTG GGATCA
CCR2	ACAGCTCAGGATTAACAGGGACTTG	ACCACTTGCATGCACA CATGAC
CCL3	GCTCAACATCATGAAGGTCTCC	TGCCGGTTTCTCTTA GTCAGG
CD86	ACGATGGACCCCAGATGCACCA	GCGTCTCCACGGAA ACAGCA
CD206	TCAGCTATTGGACGCGAGGCA	TCCGGGTTGCAAGT TGCCGT
Arg-1	CTTGCGAGACGTAGACCCTG	TCCATCACCTTGCC AATCCC
TLR4	AGGCAGCAGGTGGAATTGTATC	TCGAGGCTTTTCCATC CAATAG

### Western Blotting

Western blotting was performed according to our previous protocols (Liu et al., 2015, 2016; Qiu et al., 2016). Bilateral hippocampus (*n* = 3 in each group) were dissected and sonicated in ice cold lysis buffer (P10013B, Beyotime Institute of Biotechnology, China). The lysate was centrifuged at 12,000× *g* for 10 min at 4°C. Then the supernatant fraction was collected for Western blotting analysis. Nucleoprotein and cytoplasmic protein were extracted with Nucleoprotein and Cytoplasmic Protein Extraction Kit (KeyGEN BioTECH, China). BCA Protein Assay Kit (T9300A, Takara, Shiga, Japan) was used to detect the protein concentration. The primary antibodies used in Western blotting analysis were as follows: Rabbit Anti-Nuclear factor-κB (NFκB; P105/P50; 13586S, 1:1000; Cell Signaling, Chicago, IL, USA), Mouse Anti-NFκB (P65; 6956S, 1:1000; Cell Signaling, Chicago, IL, USA), Rabbit Anti-inhibitor of NF-κB α (IκBα; 4814S, 1:1000; Cell Signaling, Chicago, IL, USA), Rabbit Anti-P-IκBα (9246S, 1:1000; Cell Signaling, Chicago, IL, USA), Rabbit Anti-GAPDH (14C10; 2118S, 1:1000; Cell Signaling, Chicago, IL, USA). The secondary antibodies were Anti-Rabbit/Mouse IgG, HRP-linked antibody (7076/7074, 1:2000, Cell Signaling, Chicago, IL, USA). The target protein bands were quantified by using FluorChem Q system (ProteinSimple, San Jose, CA, USA).

### Immunostaining

For histological analysis, mice were anesthetized and perfused transcardially with cold phosphate buffer solution (PBS) and 4% paraformaldehyde (PFA). The whole brains were post-fixed with 4% PFA overnight and then dehydrated in 15% and 30% sucrose solutions. The brain tissues were then coated with Tissue-Tek optimal cutting temperature compound (OCT, Tissue-Tek, 4583, SAKURA, Torrance, CA, USA). All brain tissues were cut coronally into 10 μm coronal sections with Leica cryostat (CM-1950S, Leica, Germany). The slices were used for immunofluorescent staining with Ionized Calcium Binding Adapter Molecule 1 (Iba1) antibody (019-19741, 1:1000; Wako, Japan) to detect microglia. As for M1 or M2 staining, Anti-Mouse Cluster of Differentiation 86 (CD86) antibody (553689, 1:1000; BD Biosciences, San Jose, CA, USA) was used to detect M1 microglia, Mouse Macrophage Mannose Receptor (MMR)/CD206 antibody (AF2535, 1:40; R&D Systems, Minneapolis, MN, USA) was used to detect M2 microglia. For astrocytes, Glial Fibrillary Acidic Protein (GFAP) antibody (Z0334, 1:2000; Dako, Denmark) was used. The secondary antibodies were Anti-Rabbit IgG (H + L), F(ab’)_2_ Fragment (Alexa Fluor^®^ 594/488 Conjugate; 8889S/4412S, 1:2000; Cell Signaling, Chicago, IL, USA); Cy3 Goat Anti-Rat IgG (H + L; A0507, 1:2000; Beyotime, China); Alexa Fluor^®^ 555 Donkey Anti-Rabbit TgG (H + L; A0453, 1:2000; Beyotime, China) and IFKine Green, AffiniPure, Donkey Anti-Goat IgG (H + L; A24221, 1:2000; Abbkine Scientific, California, CA, USA). Pictures were visualized and photographed by a fluorescent microscope equipped with a DP80 CCD digital camera (Olympus, Tokyo, Japan). Three microscopic fields, 0.1 mm^2^ per slice were captured with the same reference position of hippocampus. The integrated density of positive staining was measured and recorded by ImageJ software on 10 slices per animal (*n* = 3 in each group).

### Statistical Analysis

All data were presented as mean ± standard error of the mean (SEM) values. Statistical significance was determined using one-way analysis of variance (ANOVA) by GraphPad Prism 7 (GraphPad Software Inc., La Jolla, CA, USA). The results were considered significant when *p*-value was less than 0.05. The *n* values in each figure legend represent the number of animals referred to the statistical analysis.

## Results

### Acute Hypoxia Induced Microglial Activation in Mouse Hippocampus

**Figure 1:**
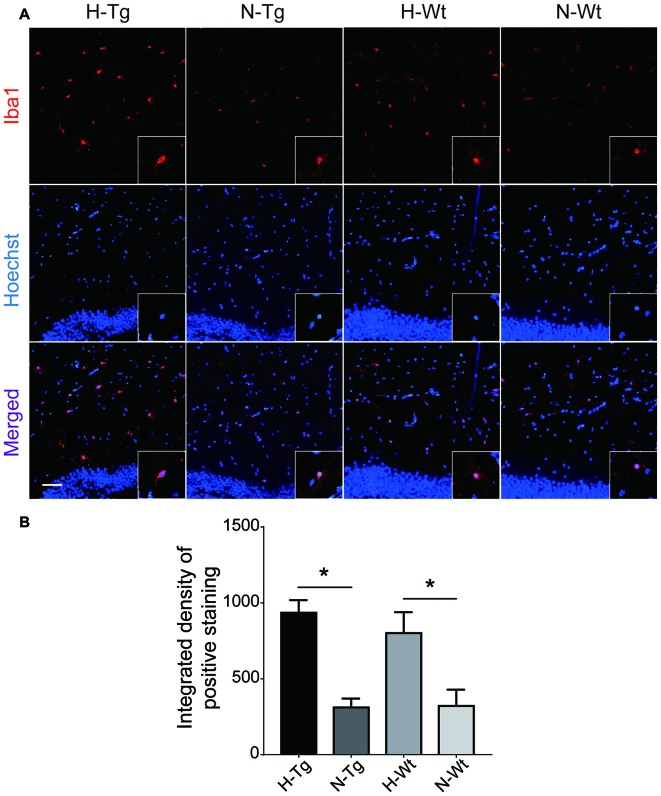
Immunoflourescence staining of microglia cells in mouse hippocampus after acute hypoxia. Microglia were detected by ionized calcium binding adapter molecule 1 (Iba1) antibody **(A)**. Iba1 positive staining was increased in H-Tg and H-Wt groups vs. N-Tg and N-Wt groups, respectively **(B)**. Scale bar: 50 μm, *n* = 3 in each group. Data were the mean ± standard error of the mean (SEM) values. *p < 0.05 by one-way analysis of variance (ANOVA).

**Figure 2:**
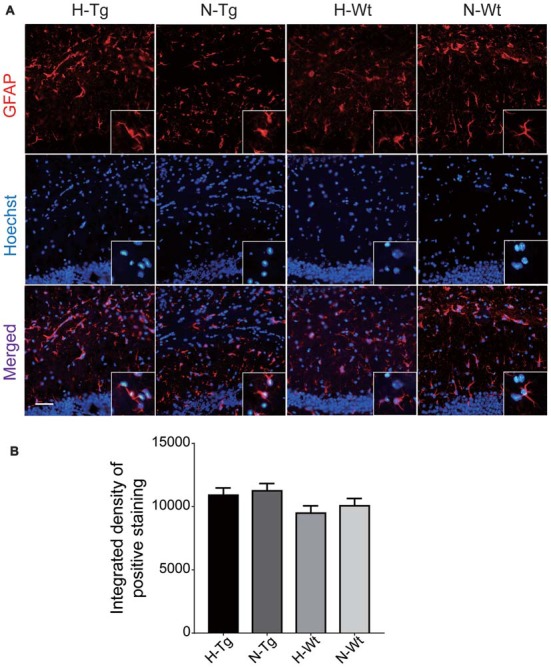
Immunoflourescence staining of astrocytes in mouse hippocampus after acute hypoxia. Astrocytes were detected by glial fibrillary acidic protein (GFAP) antibody **(A)**. The GFAP immunostaining revealed no alteration in hippocampal astrocytes after acute hypoxia exposure **(B)**. Scale bar: 50 μm, *n* = 3 in each group. Data were the mean ± SEM values.

To investigate whether acute hypoxia affected microglia activation status in AD mice, immunofluorescence staining was performed to detect microglia using anti-Iba1 antibody in hippocampus of each group. As shown in Figure 1, Iba1 positive staining increased in both H-Tg and H-Wt groups, compared with N-Tg and N-Wt groups, respectively. Quantitative analysis results showed that the integrated density in hypoxia groups increased significantly when compared to normoxia groups. Interestingly, the GFAP immunostaining revealed no alterations in hippocampal astrocytes after acute hypoxia exposure (Figure 2). All these data suggested that acute hypoxia induced microglial activation in hippocampus of both Tg and Wt mice.

### Acute Hypoxia Altered M1/M2 Phenotype in Mouse Hippocampus

**Figure 3:**
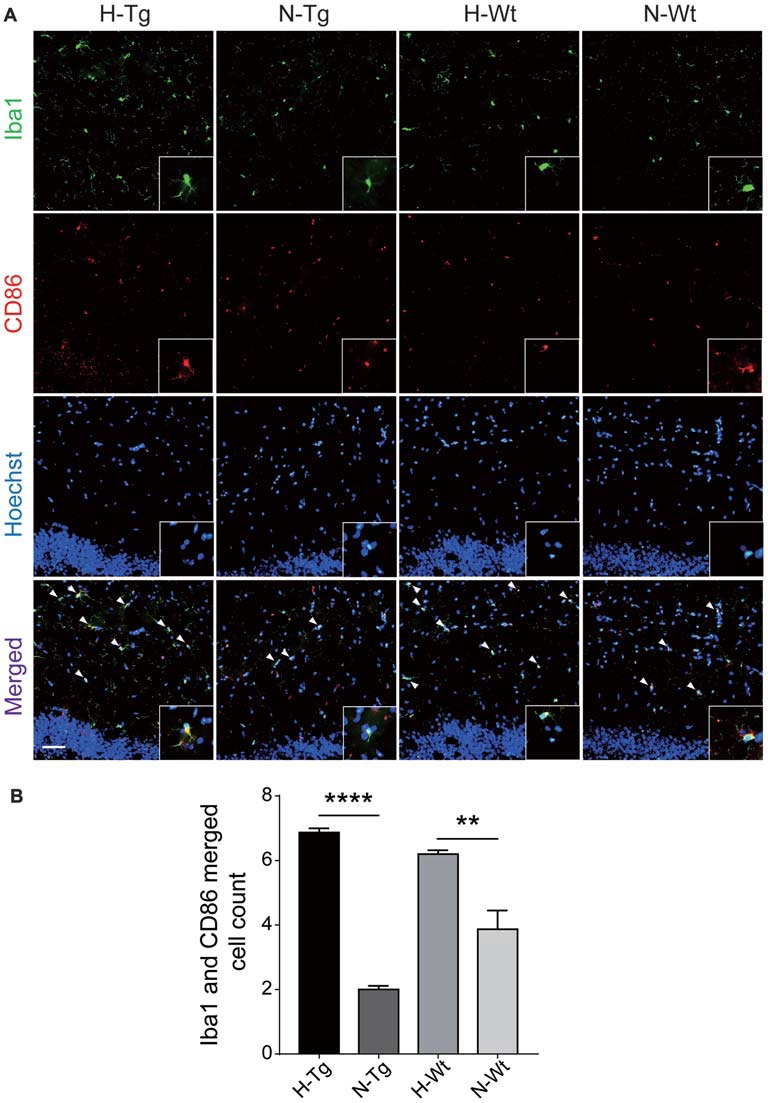
Double immunostaining of cluster of differentiation 86 (CD86; M1 marker) and Iba1 positive microglial cells in hippocampus. CD86 (red) and Iba (green) co-stained microglial cells (yellow, arrow head) were increased significantly in H-Tg and H-Wt groups vs. N-Tg and N-Wt groups, respectively **(A,B)**. Scale bar: 50 μm, *n* = 3 in each group. Data were the mean ± SEM values. **p < 0.01, ****p < 0.0001 by one-way ANOVA.

**Figure 4:**
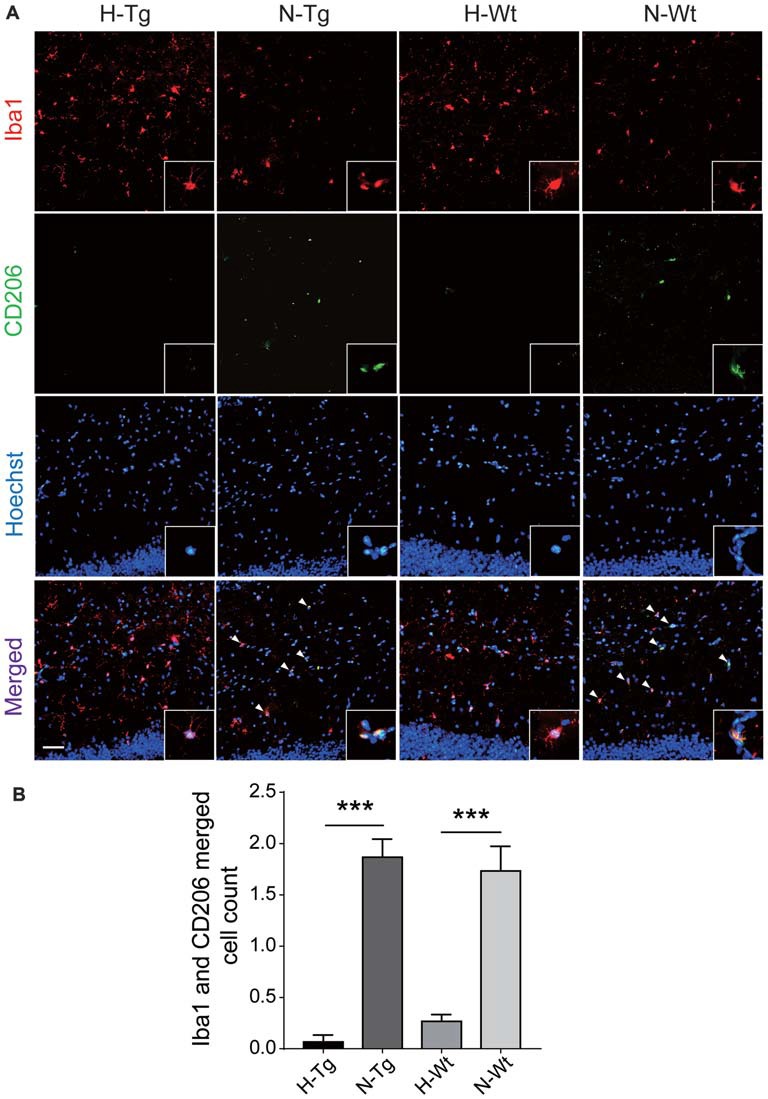
Double immunostaining of CD206 (M2 marker) and Iba1 positive microglial cells in hippocampus. CD206 (green) and Iba (red) co-stained microglial cells (yellow, arrow head) were decreased significantly in H-Tg and H-Wt groups vs. N-Tg and N-Wt groups, respectively **(A,B)**. Scale bar: 50 μm, *n* = 3 in each group. Data were the mean ± SEM values. ***p < 0.001 by one-way ANOVA.

In order to further investigate the possible impacts of acute hypoxia on hippocampal M1/M2 phenotypes transition, double staining of CD86 (M1 marker) or CD206 (M2 marker) with Iba1 was performed. The fluorescent immunostaining together with quantitative analysis clearly showed an enhanced M1 but a declined M2 phenotype of microglia, as evidenced by the increased CD86^+^/Iba1^+^ (Figure 3) and the decreased CD206^+^/Iba1^+^ (Figure 4) microglia cells, respectively.

**Figure 5:**
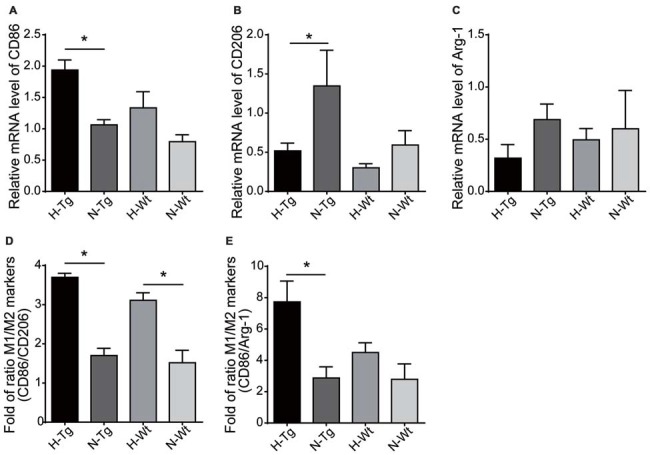
mRNA levels of M1 and M2 markers were evaluated by real-time polymerase chain reaction (PCR). M1 marker, CD86, increased in H-Tg group **(A)** and M2 markers, CD206 and arginase-1 (Arg-1), decreased in hypoxia groups **(B,C)**. The ratio of CD86/CD206 **(D)** and CD86/Arg-1 **(E)** increased significantly in hypoxia groups, *n* = 4 in each group. Data were the mean ± SEM values. *p < 0.05 by one-way ANOVA.

The mRNA levels of M1/M2 markers were further evaluated by real-time PCR. Consistent with fluorescent immunostaining, our results showed that M1 marker CD86 was increased in H-Tg group, whereas mRNA levels of M2 markers CD206 and arginase-1 (Arg-1) were decreased in H-Tg and H-Wt groups (Figure 5). Consequently, the CD86/CD206 and CD86/Arg-1 ratios increased significantly in H-Tg group (Figure 5). These data indicated that acute hypoxia enhanced M1 activation and attenuated M2 activation in hippocampus.

### Acute Hypoxia Changed the Cytokines and Chemokines Levels in Mouse Hippocampus

**Figure 6:**
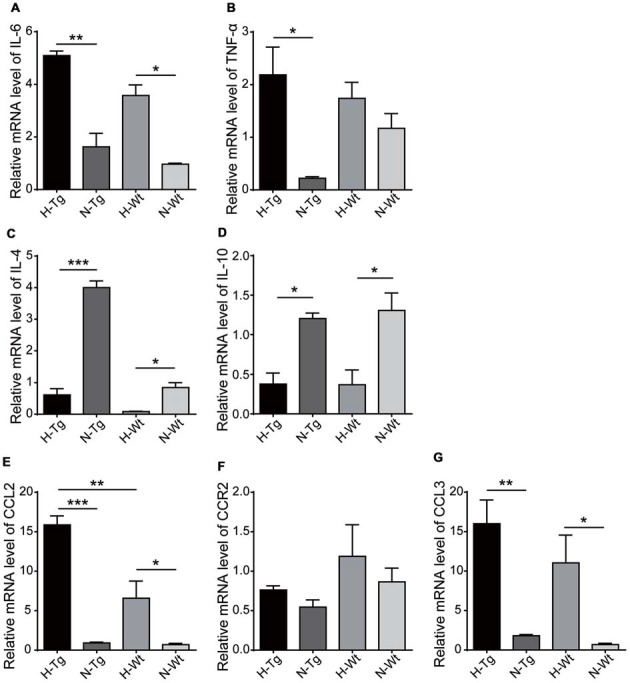
mRNA levels of cytokines and chemokines were detected by real-time PCR in hippocampus after acute hypoxic treatment. Pro-inflammatory cytokines interleukin-6 (IL-6) **(A)** and tumor necrosis factor-α (TNF-α) **(B)** increased in H-Tg group whilst anti-inflammatory cytokines IL-4 **(C)** and IL-10 **(D)** decreased in hypoxia groups. Chemokines C-C motif ligand 2 (CCL2) **(E)** and CCL3 **(G)** increased in hypoxia groups, whereas the change of C-C motif chemokine receptor 2 (CCR2) remained modest **(F)**. *n* = 4 in each group. Data were the mean ± SEM values. *p < 0.05, **p < 0.01, ***p < 0.001 by one-way ANOVA.

We next tested the mRNA levels of cytokines and chemokines in hypoxia-treated mice to confirm the above mentioned M1/M2 phenotype change. As shown in Figure 6, the relative mRNA levels of pro-inflammatory cytokines IL-6 and TNF-α were increased in H-Tg group. In contrast, the levels of two anti-inflammatory cytokines, IL-4 and IL-10, were decreased in hypoxia groups, compared to normoxia groups. These data implied that acute hypoxia induced an imbalanced M1/M2 phenotype which resulted in increased level of pro-inflammatory cytokines and decreased level of anti-inflammatory cytokines. Consistently, chemokine C-C motif ligand 2 (CCL2) and CCL3 were also increased in the mouse hippocampus of hypoxia groups. These chemokines were critical for the accumulation of activated glial cells, monocytes and lymphocytes, which might play important roles in neuroinflammation (Azizi et al., 2014).

### Acute Hypoxia Activated NF-κB Signaling in Mouse Hippocampus

**Figure 7:**
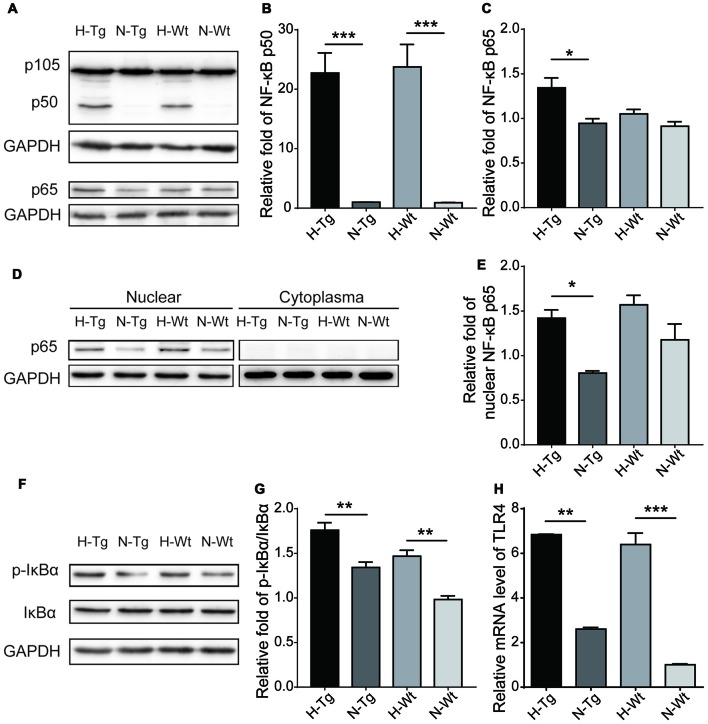
Acute hypoxic treatment activated Nuclear factor-κB (NF-κB) pathway. Protein levels of key players in NF-κB pathway were tested with western blotting. Protein levels of NF-κB p65, p50 and p-IκBα/IκBα increased significantly in hypoxia group indicating activation of NF-κB pathway **(A,B,C,F,G)**. Nucleoprotein level of NF-κB p65 increased significantly in H-Tg group **(D,E)**. mRNA level of toll-like receptor 4 (TLR4) was evaluated by real-time PCR. Significant increase of TLR4 was observed in hypoxia groups **(H)**. *n* = 3 in each group. Data were the mean ± SEM values. *p < 0.05, **p < 0.01, ***p < 0.001, by one-way ANOVA.

NF-κB family of transcription factors plays a crucial role in inflammation (Cao et al., 2006). Here, protein levels of NF-κB p50, p65 and IκBα were detected by western blotting. Our data showed that both NF-κB p50 and p65 levels were increased in H-Tg and H-Wt groups. In addition, the phosphorylation of the inhibitor of NF-κB, IκBα, increased significantly as evidenced by the increased ratio of phosphorylated IκBα (p-IκBα)/IκBα in H-Tg group, suggesting an activated NF-κB signaling pathway after acute hypoxia (Figure 7). Moreover, the nuclear NF-κB p65 was increased significantly in H-Tg group, which implied a translocation of NF-κB p65 into nucleus. Since NF-κB could be activated by toll-like receptor 4 (TLR4; Ha et al., 2011), we then investigated the relative mRNA level of TLR4 with real-time PCR. As expected, both H-Tg and H-Wt group showed a significant increase of TLR4 mRNA level.

## Discussion

Hypoxia is believed to be an important risk factor for AD, contributing to the pathological changes of Aβ and tau in AD. We have previously reported that chronic hypoxia aggravated Aβ production though epigenetic modifications of γ-secretase (Liu et al., 2016) and induced autophagy in AD mouse model (Liu et al., 2015). Other studies have confirmed that hypoxia could increase tau phosphorylation (Zhang et al., 2014; Yagishita et al., 2017). Furthermore, our group and others have demonstrated that hypoxia can significantly activate microglia which is believed to play an important role in the pathogenesis of AD (Zhang et al., 2013; Sapin et al., 2015). It is known that pro-inflammatory cytokines released by microglia can increase Aβ production and decrease its clearance (Cai et al., 2014). Persistent increase of pro-inflammatory cytokines and chronic activation of microglia may cause chronic inflammatory status leading to neuronal damage and neurodegeneration (Calsolaro and Edison, 2016). With more knowledge of the interplay among hypoxia, neuroinflammation and AD pathogenesis, anti-inflammatory treatment is likely to be successful in AD. And more therapeutic targets could be found in studies of mechanisms of hypoxia-induced neuroinflammation.

Microglia can be activated as M1 and M2 phenotypes. The pro-inflammatory M1 phenotype predominates at the site of neuroinflammation and is associated with the release of pro-inflammatory cytokines and chemokines which may cause cell death and tissue damage. In contrast, M2 microglial phenotype appears later and is related to repair processes with anti-inflammatory property (Bolós et al., 2017). Previous study has reported that short-term hypoxia could increase the expression of pro-inflammatory cytokines and favored M1 activation of microglia *in vitro*. An increased iNOS and decreased Trem2 and Arg-1 have been reported in cultured primary rat microglia after 3-h hypoxia (Habib et al., 2014). Another study of neonatal hypoxic-ischemic brain injury in mice showed that CD86 positive cells were increased and relative proportion of CD206 positive cells were reduced after injury, indicating that hypoxia might facilitate M1 polarization and attenuate M2 activation (Hellström Erkenstam et al., 2016). Consistently, in current study, we found that acute hypoxia increased M1 marker CD86 and reduced M2 markers, CD206 and Arg-1, along with the increased levels of pro-inflammatory cytokines and the reduced levels of anti-inflammatory cytokines in hippocampus of AD mouse model.

Moreover, the levels of chemokines, CCL2 and CCL3, increased significantly in hypoxia groups. CCL2, also known as monocyte chemoattractant protein 1 (MCP1), is a chemokine produced by neurons and glial cells and induces chemotaxis of monocytes and microglia, which contributes to the pathological microgliosis (Westin et al., 2012). Activated monocytes recruited into the brain are further differentiated into macrophages producing neurotoxic molecules (Azizi et al., 2014). Previous study reported that CCL2 overexpression induced microglial accumulation and facilitated Aβ oligomer formation, resulted in an enhanced plaque formation and accelerated memory deficits in APP/CCL2 bigenic mice (Kiyota et al., 2009). CCL2 overexpression was also reported to elevate the expression of apolipoprotein E and thus increase Aβ deposition by reducing the clearance (Yamamoto et al., 2005). CCL3 or human macrophage inflammatory protein 1α (MIP-1α) is a member of β-chemokine subfamily and is involved in the recruitment and activation of polymorphonuclear leukocytes. Previous study found that AD patients had a higher level of CCL3 in peripheral T lymphocytes compared to age-matched healthy controls (Man et al., 2007). CCL3 induced the expression of CCR5, a potential receptor of CCL3, on brain microvascular endothelial cells constituting the blood-brain barrier and resulted in an increased T cells transendothelial migration from blood to the brain (Man et al., 2007). Then, the accumulated T cells might lead to the increased levels of pro-inflammatory cytokines and cause chronic inflammation, which enhanced neurotoxicity and impaired functions of microglia (Mietelska-Porowska and Wojda, 2017). T cell infiltrating the brain might also contribute to the cognitive impairment of tau pathology. One recent study found that hippocampal T cells might modulate microglial and/or astrocytic activation status and lead to detrimental impact on synaptic plasticity (Laurent et al., 2017).

NF-κB family of transcription factors plays a crucial role in inflammation, immunity and cell proliferation (Viatour et al., 2005). Activation of NF-κB pathway is triggered by a variety of extracellular stimuli and recruited IκB-kinase (IKK) complex. Once activated, the IKK complex phosphorylates IκB proteins, which leads to the proteasome-mediated degradation of IκB proteins and allows the NF-κB to translocate to the nucleus to execute transcription of target genes (Viatour et al., 2005; Kawai and Akira, 2007). In the present study, we further investigated protein levels of key players in NF-κB pathway and found that both NF-κB p50 and p65 subunit and nucleoprotein level of NF-κB p65 increased in hypoxia groups with a higher ratio of p-IκBα/IκBα, indicating that the increased pro-inflammatory cytokines and chemokines might be associated with activated NF-κB pathway. TLR4 is a type-I transmembrane receptor expressed in neurons, astrocytes and microglia. Its activation is involved in microglia-mediated inflammation responding to many insults and leads to the production and release of cytokines including IL-1β, IL-6, TNF-α and iNOS (Smith et al., 2013). NF-κB is one of the most important downstream transcription factors in TLR signaling pathways. TLR4 signaling induces the phosphorylation of IκB and the subsequent degradation, which promotes the nuclear translocation of NF-κB, stimulating the transcription of various target genes (Ha et al., 2011). Previous studies showed that chronic intermittent hypoxia activated NF-κB pathway through TLR4 signaling and might contribute to hippocampal neuronal damage (Smith et al., 2013; Deng et al., 2015). In our study, we found an increased mRNA level of TLR4 in acute hypoxia groups which might indicate that acute hypoxia could activate NF-κB pathway through TLR4 signaling.

In summary, here we investigated microglia activation and cytokines levels in hippocampus of APP/PS1 mouse model after acute hypoxic treatment. We found that acute hypoxia favored M1 activation and attenuated M2 activation, which resulted in the release of pro-inflammatory cytokines and chemokines, such as IL-6, TNF-α, CCL2 and CCL3, and contributed to the pathogenesis of AD. Acute hypoxia induced activation of microglia might be associated with the activation of NF-κB pathway through TLR4 signaling.

## Author Contributions

WL designed the project of this manuscript; FZ, RZ and ZF carried out all the experiments. FZ, RZ, SL and CC contributed to statistical analyses and results interpretation. FZ, RZ, ZF and SL contributed to drafting of the manuscript. RZ, SL, HC and WL revised the manuscript. WL contributed to research concept, research administration. All authors edited and approved the final version of the manuscript.

## Conflict of Interest Statement

The authors declare that the research was conducted in the absence of any commercial or financial relationships that could be construed as a potential conflict of interest.
